# Imposter phenomenon among health professionals and students: A protocol for systematic review and meta analysis

**DOI:** 10.1097/MD.0000000000034364

**Published:** 2023-07-21

**Authors:** Mohammad Azmain Iktidar, Rifat Ara, Simanta Roy, Mashrur Ahmed, Sreshtha Chowdhury, Fahima Nasrin Eva, Sadia Mahmud Trisha, Azaz Bin Sharif

**Affiliations:** a Department of Public Health, North South University, Dhaka, Bangladesh; b School of Research, Chattogram, Bangladesh; c International Centre for Diarrheal Disease Research Bangladesh, Dhaka, Bangladesh.

**Keywords:** imposter, impostor, burnout, imposterism

## Abstract

Health professionals and medical students are at increased risk of the imposter phenomenon (IP) in other words, the imposter syndrome, due to the challenging nature of their professions. It is particularly concerning since it is linked to a higher incidence of burnout and suicidal ideation. We must first thoroughly grasp its prevalence and associated factors to address this issue. However, no published review of the data includes a meta-analysis to help understand the character and associated factors of IP among medical workers and medical students. This study aims to investigate IP prevalence and risk factors among healthcare personnel. Five online databases will be searched for papers published in English between January 2000 and December 2022, and 2 independent reviewers will filter, select studies, extract data, and evaluate the risk of bias in each piece. The retrieved articles will be included based on strict inclusion and exclusion criteria. A third reviewer will decide on any disagreements between the 2 reviewers. Where appropriate, a meta-analysis will be conducted using the random-effects model. The heterogeneity of the studies will be examined, and a sensitivity analysis will be done depending on the study quality. The purpose of this review is to determine the prevalence and risk factors for IP among healthcare personnel. The review’s findings will emphasize the severity and contributing factors of the problem, therefore guiding policy for future actions.

## 1. Introduction

The imposter phenomenon (IP), in other words, the imposter syndrome, was first defined by Clance and Imes in 1978. It is referred to as the inability to internalize achievement and a tendency to attribute success to external factors such as luck, error, or personal connections.^[1,2]^ The imposter phenomenon may cause psychological distress, emotional suffering, and significant mental health issues, such as persistent dysphoric stress, anxiety, depression, and drug dependence, on an individual level.^[3]^ Even though imposter syndrome is not a recognized clinical illness nor a psychiatric disease in the “Diagnostic and Statistical Manual of Mental Disorders,” psychologists and others acknowledge the existence of a very realistic and unique kind of formal self-doubt.^[4]^ Although Clance and Imes^[1,2]^ first used the name “impostor phenomena,” academic studies and social media commonly refer to it as the “impostor syndrome,” which emphasizes the phenomenon’s apparent individuality and dysfunction.^[5–7]^ But the term “syndrome is used to describe the condition, which provides the sense that those who experience it are “patients.”^[5]^ This is very problematic since it indicates a medical model of the person’s internal dysfunction.^[8]^ Hence, the word “phenomenon” is preferred by several researchers.

The research on the imposter phenomenon impacts has seen a renaissance in recent years. However, there still needs to be more clarity about the condition’s underlying causes. Even though Clance and Imes’ initial research focused on women, imposter phenomenon characteristics may be seen in men and women.^[1]^ It was previously thought to afflict just a tiny number of people, and newer studies have shown that it affects both men and women and people from a wide range of diverse ethnic and cultural backgrounds.^[9–12]^ It is common for people to feel fraudulence when they achieve personal or professional success. This phenomenon is referred to as impostorism.^[1]^

Some think that ambiguous parental signals of overpraise and criticism and familial pressure to achieve at an early age may have contributed to dishonest sentiments in these adult children. Additionally, it has been shown that first-generation college students and those who attain fast success are more likely to have experiences of self-doubt.^[13]^ Many students who have imposter emotions suffer in silence because they are hesitant or embarrassed to discuss their opposing ideas. Although imposter syndrome has been adequately documented in other disciplines,^[14,15]^ medicine has a somewhat limited grasp of the phenomena. This is especially concerning given the high expectations and perfectionism prevalent in this area, which puts persons at considerable risk for having IP. Burnout affects around 67% of practicing doctors and 76% of residents, according to recent research.^[16–18]^ According to one study, high rates of imposter phenomenon among doctors lead to burnout and early retirement and may contribute to increased physician suicide rates.^[19]^ In another study by Mattie et al, 30% of the total medical assistant who participated had imposter sentiments about themselves^[20,21]^

Imposter phenomenon has gotten much attention outside of academics, particularly in the context of professional success. Although there has been a qualitative study,^[21]^ there have been very few published systematic reviews of the studies on imposter phenomenon, which may be because it is not an acknowledged medical condition, even though there is a lot of peer review and lay literature about it. The purpose of this report was to conduct a comprehensive review of the existing literature on imposter phenomenon, with a focus on determining the prevalence of imposter phenomenon among healthcare workers and characterizing the associated factors.

## 2. Methods

### 2.1. Protocol

The procedure for this systematic review adheres to the accepted standards for systematic reviews as defined by PRISMA-P (Preferred Reporting Items for Systematic Reviews and Meta-Analyses Protocol) and the Cochrane group.^[22]^ The supplementary file contains a PRISMA-P checklist. This review’s protocol has been registered with the International prospective register of systematic reviews – PROSPERO network (Reg. number: CRD42021291207).

### 2.2. Eligibility criteria

Peer-reviewed studies will be eligible for inclusion. Cross-sectional surveys, longitudinal studies involving healthcare professionals and medical students in different hospitals, medical institutions will be considered for this review. Studies used the CIPS for determining the presence of imposter phenomenon will be included.^[23]^

Letters to the editor, editorials, and conference papers will not be accepted. Also omitted will be articles written in languages other than English. Only dissertations, scale validations, reported dementia- or delirium-based illnesses, or published cases of legal fraud, imposter medicines, Munchausen’s, or Munchausen’s via proxy would be omitted.

### 2.3. Participants

Healthcare professionals who are already graduates and also current students of medical, dental, nursing, pharmacy, psychology units will be considered as our study population. Based on this population criteria, individuals who were suffering from imposter phenomenon and participated in the primary study will be our targeted participants.

### 2.4. Interventions and comparators

No interventions will be considered, as this review does not include any randomized controlled trial and will be looking only at the prevalence of the imposter phenomenon among health professionals and students worldwide.

No comparators will be considered in this review as there will be no RCTs encompassed in this review, but the prevalence of imposter phenomenon will be compared among different gender and health professional groups.

### 2.5. Outcomes

Imposter syndrome, imposter phenomenon, or Impostorism, will be our review’s primary outcome. Correlation of imposter syndrome with different variables, such as gender, demographic variables, personality, and workplace outcomes, will be considered our additional outcomes. Measures of the effect of these outcomes will be the prevalence rate, odds ratio (OR), or relative risk (RR), as mentioned in the included articles. It will be expressed at least one-time point (or multiple if available), as mentioned in the included studies.

### 2.6. Information sources

The systematic review will be based on citations from January 2000 to December 2022 in databases such as MEDLINE, PsycINFO, Web of Science, Scopus, Google Scholar, and the Cochrane Database of Systematic Reviews. Search phrases will be tailored for use in various bibliographic databases in combination with database-specific filters.

### 2.7. Search strategy

A thorough search strategy for all above mentioned sources will be created. Where available, the search phrases will be altered for use with other bibliographical databases via database-specific filters. The essential search keywords for population, intervention, comparison, and outcomes are summarized in Table 1. The search will be limited to English-language literature. The search will look for studies published between January 2000 to December 2022. Before the final analysis, the searches will be redone, and additional studies will be obtained for consideration. Table 2 contains a complete search strategy for Pubmed.

**Table 1:** Essential search keywords for PICO (population, intervention, comparison, and outcomes).

Population	Intervention	Comparison	Outcome
Health care worker	No intervention is applicable for this review.	Prevalence	Imposter phenomenon
OR		OR	OR
Doctor		Epidemiology	
OR			Impostor phenomenon
Physician		**AND**	OR
OR			Imposter
Medical student		Risk factors	syndrome
OR		OR	OR
Resident physician		Associated factors	Impostor
OR		OR	syndrome
Attending physician		Factors	OR
OR		OR	Impostorism
Nurse		Threatening factors	
OR		OR	
Nursing student		Causes	
OR			
Pharmacist			
OR			
Pharmacy student			
OR			
Medical graduate			
OR			
Medical consultant			
OR			
Intern			
OR			
Dentist			
OR			
Dental student			
OR			
Dental surgeon			

**Table 2:** A complete search strategy for Pubmed.

1.	“health personnel”[MeSH Terms] OR “health personnel”[All Fields] OR “health care worker”[All Fields] OR (“doctor s”[All Fields] OR “doctoral”[All Fields] OR “doctorate”[All Fields] OR “doctorates”[All Fields] OR “doctoring”[All Fields] OR “doctor”[All Fields] OR “doctors”[All Fields]) OR (“physician s”[All Fields] OR “physicians”[MeSH Terms] OR “physicians”[All Fields] OR “physician”[All Fields] OR “physicians s”[All Fields]) OR (“students, medical”[MeSH Terms] OR (“students”[All Fields] AND “medical”[All Fields]) OR “medical students”[All Fields] OR (“medical”[All Fields] AND “student”[All Fields]) OR “medical student”[All Fields]) OR (“internship and residency”[MeSH Terms] OR (“internship”[All Fields] AND “residency”[All Fields]) OR “internship and residency”[All Fields] OR “resident”[All Fields] OR “resident s”[All Fields] OR “residents”[All Fields] OR “attending physician”[All Fields] OR (“nurse s”[All Fields] OR “nurses”[MeSH Terms] OR “nurses”[All Fields] OR “nurse”[All Fields] OR “nurses s”[All Fields]) OR (“students, nursing”[MeSH Terms] OR (“students”[All Fields] AND “nursing”[All Fields]) OR “nursing students”[All Fields] OR (“nursing”[All Fields] AND “student”[All Fields]) OR “nursing student”[All Fields]) OR (“pharmacist s”[All Fields] OR “pharmacists”[MeSH Terms] OR “pharmacists”[All Fields] OR “pharmacist”[All Fields]) OR (“students, pharmacy”[MeSH Terms] OR (“students”[All Fields] AND “pharmacy”[All Fields]) OR “pharmacy students”[All Fields] OR (“pharmacy”[All Fields] AND “student”[All Fields]) OR “pharmacy student”[All Fields]) OR “medical graduate”[All Fields] OR “medical graduates”[All Fields] OR “consultants”[MeSH Terms] OR “consultants”[All Fields] OR “medical consultants”[All Fields] OR “consulter”[All Fields] OR “consulters”[All Fields] OR “referral and consultation”[MeSH Terms] OR (“referral”[All Fields] AND “consultation”[All Fields]) OR “referral and consultation”[All Fields] OR (“intern”[All Fields] OR “intern s”[All Fields] OR “internes”[All Fields] OR “interning”[All Fields] OR “interns”[All Fields]) OR (“dentist s”[All Fields] OR “dentists”[MeSH Terms] OR “dentists”[All Fields] OR “dentist”[All Fields]) OR (“students, dental”[MeSH Terms] OR (“students”[All Fields] AND “dental”[All Fields]) OR “dental students”[All Fields] OR (“dental”[All Fields] AND “student”[All Fields]) OR “dental student”[All Fields]) OR “dentists”[MeSH Terms] OR “dentists”[All Fields] OR (“dental”[All Fields] AND “surgeon”[All Fields]) OR “dental surgeon”[All Fields])
2.	“epidemiology”[MeSH Subheading] OR “epidemiology”[All Fields] OR “prevalence”[All Fields] OR “prevalence”[MeSH Terms] OR “prevalance”[All Fields] OR “prevalences”[All Fields] OR “prevalence s”[All Fields] OR “prevalent”[All Fields] OR “prevalently”[All Fields] OR “prevalents”[All Fields] OR “epidemiologies”[All Fields] OR “epidemiology”[MeSH Terms] OR “epidemiology s”[All Fields]
3.	“risk factors”[MeSH Terms] OR (“risk”[All Fields] AND “factors”[All Fields]) OR “risk factors”[All Fields] OR “associated factors”[All Fields] OR “associating factors”[All Fields] OR (“association”[MeSH Terms] OR “association”[All Fields] OR “associations”[All Fields]) OR (“factor”[All Fields] OR “factor s”[All Fields] OR “factors”[All Fields]) OR (“threaten”[All Fields] OR “threatened”[All Fields] OR “threatening”[All Fields] OR “threatens”[All Fields]) OR (“causative”[All Fields] OR “causatively”[All Fields] OR “causatives”[All Fields] OR “cause”[All Fields] OR “caused”[All Fields] OR “causing”[All Fields] OR “etiology”[MeSH Subheading] OR “etiology”[All Fields] OR “causes”[All Fields])
4.	“imposter syndrome”[Supplementary Concept] OR “imposter syndrome”[All Fields] OR “impostor phenomenon”[All Fields] OR “impostor”[All Fields] OR “impostorism”[All Fields] OR “impostors”[All Fields]
5.	1 AND 2 AND 3 AND 4

In terms of the dearth of sufficient journal articles, other sources (Scopus, Cochrane Database of Systematic Reviews, PsycINFO) will be searched. Additionally, the search strategy will be reinforced by (a) analyzing citations in the reference lists of included papers and related systematic reviews, as well as (b) using Google Scholar. Table 3 contains a complete search strategy for Scopus.

**Table 3:** A complete search strategy for Scopus.

1.	ALL (“Prevalence” OR “Epidemiology” AND “Risk factors” OR “Associated factors” OR “Factors” OR “Threatening factors” OR “Causes” AND “Impostor phenomenon” OR “impostor syndrome” OR “Impostorism”)
2.	ALL (“Prevalence” OR “Epidemiology” AND “Risk factors” OR “Associated factors” OR “Factors” OR “Threatening factors” OR “Causes” AND “Impostor phenomenon” OR “impostor syndrome” OR “Impostorism”) AND PUBYEAR > 1999
3.	ALL (“Prevalence” OR “Epidemiology” AND “Risk factors” OR “Associated factors” OR “Factors” OR “Threatening factors” OR “Causes” AND “Impostor phenomenon” OR “impostor syndrome” OR “Impostorism”) AND PUBYEAR > 1999 AND (LIMIT-TO (DOCTYPE, “ar”) OR LIMIT-TO (DOCTYPE, “cp”)) AND (LIMIT-TO (LANGUAGE, “English”))

### 2.8. Data screening and extraction

Two impartial reviewers will undertake a preliminary screening of the title and abstract following the inclusion and exclusion criteria. Relevant articles will be outlined and included in the evaluation process. Rayyan QCRI software will be used for screening. If there is any doubt or dispute amongst the reviewers over the selection at this point, the third reviewer will settle it. Repeated studies and various journal articles from the same studies will be omitted, with all exemption justifications acknowledged. The PRISMA flow diagram will illustrate how publications were identified and included for review using specific evaluation criteria. A standard form will be constructed using Excel Spreadsheet to retrieve data from the listed papers. JBI critical appraisal checklist will be used to evaluate the study’s quality.^[24]^ The following information will be extracted: study setup; study people; participant demographics and baseline characteristics; study methodology; selection procedure and completion rates; findings and assessment times; markers of acceptability to users; data for assessing the risk of bias, etc.

### 2.9. Risk of bias assessment

The risk of bias of the included studies will be assessed independently by 2 reviewers using the Cochrane Risk of Bias assessment tools will be accomplished via the use of 7 evidence-based domains: random sequence generation, masking of allocation, blinding of subjects and supervisors, blinding of evaluation assessment, incomplete outcome data, selective reporting, and other bias. Based on the review author’s judgments, they will be classified as low, moderate, or high risk of bias. Disagreements between the reviewers will be discussed and resolved by consensus. However, insights from the third reviewer will be sought if necessary.

### 2.10. Strategy for data synthesis

Following a comprehensive text screening of the publications, the prevalence rate will be measured and tabulated. A brief overview will be provided regarding publication time and study area. A narrative synthesis of the included papers will be conducted, concentrating on the targeted population and the result since this enables comparisons and categorization of the chosen homogeneous research. For dichotomous results, the effect size will be expressed as an OR or RR with a 95% confidence interval (CI), whereas for continuous data, the mean difference (MD) with a 95% CI will be expressed and considered in conjunction with the standardized mean difference (with 95% CI) in the case of identical outcomes measured on multiple scales. The χ^2^ test and the *I*^²^ statistics will examine the studies’ heterogeneity in terms of effective measures. Additionally, sensitivity analysis will be performed dependent on the study’s quality. A funnel plot will be developed for each paper using the review management software (RevMan) to analyze possible publication bias.

### 2.11. Analysis of subgroups or subsets

If data is accessible, subgroup analysis for variables such as race, gender, and job status will be performed and incorporated in meta-regression models to assess the influence of participant characteristics on this syndrome.

## 3. Discussion

Although the original finding by Clance and Imes^[1]^ focused on women academics in high level positions, it is evident that IP tendencies occur in people from all walks of life. It is predicted that three-quarters of all people will be affected by it at some point throughout their lives.^[13]^ This prevalence is staggeringly high (30%) among healthcare students, as reported by Mattie et al.^[20]^ Another study discovered that early retirement due to a high burnout rate in physicians could be linked to a high prevalence of imposter phenomenon. This finding also suggests imposter phenomenon can determine the rising suicide rate among physicians.^[19]^

This systematic review also complies with the sustainable development goals, since sustainable development goals 3.4 targets “one-third reduction in premature mortality from non-communicable diseases through prevention and treatment and promote mental health and well-being.”^[25]^ Therefore, it is high time to appraise the prevalence of imposter phenomenon among health professionals and students. Considering that more and more research is being conducted in this area, a systematic review with meta-analysis is missing to summarize the results on the prevalence rates of the IP among health professionals and students. By presenting a quantitative synthesis, it is possible to identify gaps in the existing literature and recommend areas for further investigation. This review will compile the empirical evidence of the prevalence of IP among healthcare professionals and students, so it may assist the government or donor organizations to determine future financing and policy-making decisions.

This review will follow a comprehensive methodology to sift through and consolidate available literature. In addition, each study will be evaluated for quality, and the results will be summarized in a table. While this is beyond our control, this systematic review is constrained by its dependence on published data; consequently, publication bias cannot be ruled out, as unpublished findings are not included in the review. This review will include only publications written in the English language; as a result, we may overlook evidence published in other languages. Although this study will follow a systematic and transparent approach, it will also require subjective assessments by review authors, which may introduce bias.

## Amendments

Any changes or revisions to this protocol will be included in a table together with the date, a description of the change, and the reason for the change. The protocol and any revisions will be added to the International Prospective Register of Systematic Reviews.

## Author contributions

**Conceptualization:** Mohammad Azmain Iktidar, Rifat Ara, Simanta Roy.

**Data curation:** Simanta Roy.

**Methodology:** Rifat Ara, Simanta Roy, Mashrur Ahmed.

**Project administration:** Mohammad Azmain Iktidar, Simanta Roy, Azaz Bin Sharif.

**Supervision:** Rifat Ara, Azaz Bin Sharif.

**Writing – original draft:** Mohammad Azmain Iktidar, Mashrur Ahmed, Sreshtha Chowdhury, Fahima Nasrin Eva, Sadia Mahmud Trisha.

**Writing – review & editing:** Sreshtha Chowdhury, Fahima Nasrin Eva, Sadia Mahmud Trisha, Azaz Bin Sharif.
